# An interdisciplinary, co-designed guide for return to running postpartum—a mixed-methods study

**DOI:** 10.3389/fspor.2026.1771882

**Published:** 2026-03-30

**Authors:** Megan L. James, Diane M. Crone, Lynne Evans, Victoria H. Stiles, Gráinne M. Donnelly, Isabel S. Moore

**Affiliations:** 1 Cardiff School of Sport and Health Sciences, School of Sport and Health Sciences, Cardiff Metropolitan University, Cardiff, United Kingdom; 2 Centre for Health, Activity and Wellbeing Research (CAWR), Cardiff Metropolitan University, Cardiff, United Kingdom; 3 Department of Public Health and Sport Sciences, University of Exeter, Exeter, United Kingdom

**Keywords:** childbirth, guidance, mother, pregnancy, runner

## Abstract

**Introduction:**

Despite its ease of access and multifaceted health benefits, compared to pre-pregnancy, engagement in running decreases following childbirth (postpartum). While a biopsychosocial approach is recommended to help women return to running postpartum, greater understanding of running-specific barriers and facilitators is needed to support this. To date, there is a lack of evidence-based, co-designed guidance to help facilitate re-engagement with running postpartum. The aims of this study were therefore to: 1) investigate the barriers to and facilitators of postpartum return to running; and 2) co-design a postpartum return to running intervention that is underpinned by clinical recommendations.

**Methods:**

The study adopted the iterative Double Diamond co-design framework to “Discover” and “Define” the problem and then “Develop” solutions to the problem. First, barriers and facilitators of return to running, as well as considerations for a postpartum return to running intervention, were identified through an e-survey and follow-up focus groups (“Discover”). The findings from the “Discover” phase were used to “Define” the problem and identify initial solutions for a postpartum return to running intervention. The research team and an Advisory Group of postpartum runners then co-designed the intervention (“Develop”).

**Results:**

Four themes were identified regarding what needs to be considered in a return to running intervention: a) fitting it in, b) physical considerations, c) psychosocial considerations, and d) external considerations. Participants highlighted their negative psychological and physical experiences of pelvic floor dysfunction and desire for pregnancy- and postpartum-specific graded rehabilitation. Following iterative co-design with an Advisory Group of postpartum runners, a biopsychosocial, person-centred guide for return to running was developed.

**Discussion:**

This study culminated in the co-design of a biopsychosocial, clinically endorsed guide for postpartum return to running. Future research should aim to test the feasibility and effectiveness of the guide (“Deliver”), which, it is hoped, may foster increased re-engagement with running postpartum in the future.

## 1 Introduction

Engaging with physical activity and exercise following childbirth (postpartum) is associated with many biopsychosocial health benefits (1–6). Although running provides an accessible, low-cost form of physical activity, some mothers who decrease their engagement with running during pregnancy do not return to running postpartum at all (7–9). The 6Rs framework is a six-phase return to sport postpartum framework that advocates a sport-specific, individualised, biopsychosocial approach; from preparation prior to birth, to return to sport and performance (10). However, to adopt this framework, greater insight into the distinct running-related barriers and facilitators postpartum women experience is needed to ensure it accounts for cohort-specific needs.

Studies to date have largely focussed on factors affecting engagement in postpartum physical activity and exercise generally, as opposed to return to running specifically (11–13). Factors such as a lack of time, energy/sleep, confidence, motivation and postpartum depression have been identified as reasons for general physical inactivity postpartum (12–16). Facilitators to overcome such barriers include having structured exercise classes, available childcare and increased partner and social support (12, 13, 17). The small number of studies that have examined factors affecting running specifically, have almost exclusively focussed on the barriers to, rather than facilitators of running (7, 8, 18). In these studies, reasons for not returning to running include pelvic floor dysfunction symptoms, a fear of movement, fatigue and pain and a lack of confidence and advice (7, 8). While these studies have provided some important preliminary insights, they did not adopt a user-centred approach and were undertaken during COVID-19; potentially limiting their reach and significance. In addition, an increased understanding of facilitators for return to running is needed to help remove barriers for those who have failed to return to running. Further, despite the online release of the first consensus-based clinical guidelines into the public domain, to address the lack of advice available regarding return to running postpartum (19), only 35% of postpartum runners in an international, online survey study were aware they existed (18). These guidelines need to be further developed with mothers, evidence-informed using systematic and reproducible methods, and more widely publicised, to increase the likelihood of postpartum return to running (18).

Current guidance for return to running postpartum does not thoroughly account for biopsychosocial factors and is informed by, and predominantly targeted at, clinicians rather than postpartum runners [see (19, 20)]. One way in which research may be better tailored to end-users is through stakeholder engagement (21). Approaches like co-design, co-production, and patient and public involvement are informed by end-users’ lived experience and have become increasingly employed for intervention development with the perinatal population (22–24) to increase the efficacy of intervention design (25). The Double Diamond framework (26) offers a basis for co-design and has been used in a range of contexts, including physical activity promotion (27, 28). However, to the authors’ knowledge, no co-designed interventions for return to running postpartum currently exist. The aims of this study were therefore to: 1) investigate the barriers to and facilitators of postpartum return to running; and 2) co-design a postpartum return to running intervention that is underpinned by clinical recommendations.

## 2 Materials and methods

### 2.1 Study design

**Figure 1:**
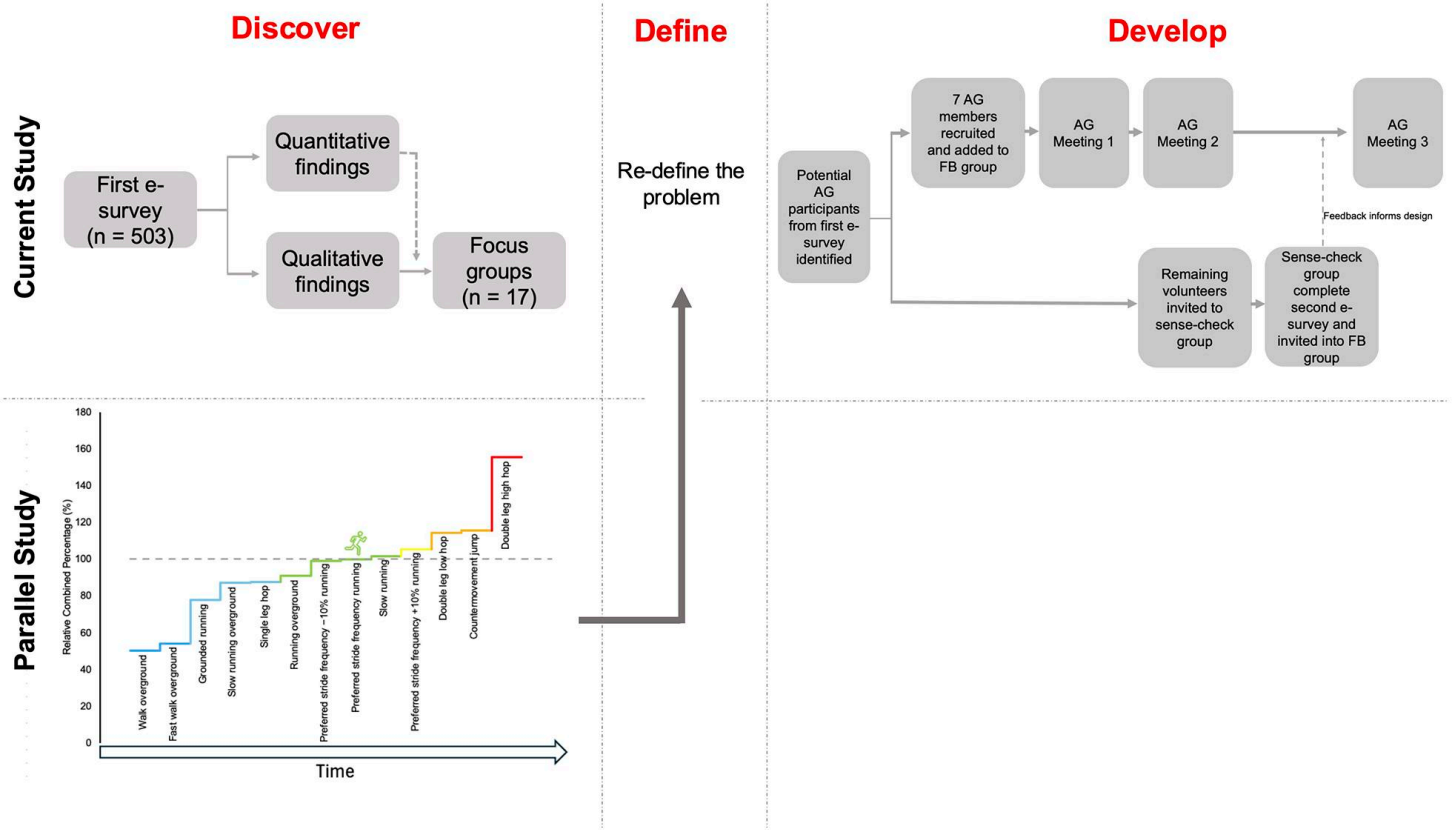
Phases of this study and findings from a parallel study, the physical “programme” (33), that inform the co-design of a return to running postpartum intervention, aligned to stages of the double diamond framework. The “Deliver” phase was beyond scope of this study. AG = Advisory group, FB = Facebook group. The relative combined percentage in the parallel study represents a mean of four pelvic acceleration variables, measured with an inertial measurement unit, captured during each activity (where normal treadmill running = 100%). This parallel, exploratory study was not conducted with a postpartum sample; however, this is a recommendation for future research; see (33) for further information.

This study adopted a mixed-methods, co-design approach, underpinned by the Double Diamond framework (26). The Double Diamond framework consists of a four-step process, separated into two diamonds, promoting divergent (widely considering issues) and convergent (more focussed) thinking (26). Figure 1 presents the stages of the study aligned with these phases of the Double Diamond framework. The “Discover”, “Define”, and “Develop” phases will be outlined in the following Methods, Results and Discussion sections. The final phase of the Double Diamond framework, “Deliver”, is where testing of the solution or intervention begins. This stage was beyond the scope of this study and is a recommendation for future research.

### 2.2 Discover

The first phase of the Double Diamond (“Discover”) ensures the problem is understood and involves speaking to problem-owners/end-users. To gain further initial insights, prior to speaking to end-users, a literature review was undertaken. This identified that more understanding of biopsychosocial factors affecting the return to running postpartum was needed. It was also concluded that a graded programme for return to running postpartum that is specific to the pelvis may be beneficial to address factors such as fear of movement, confidence and stress urinary incontinence (29–32). Finally, co-designed recommendations for return to running were needed to address the current lack of guidance available for return to running postpartum, and meet the specific needs and wants of those that require them (21). These findings informed the subsequent research activities. The rest of the “Discover” phase included obtaining the input of end-users via an e-survey (e-survey_1_) and follow-up focus groups, while an additional parallel biomechanics study informed the physical ‘programme’ aspect of the intervention [see (33), Figure 1].

#### 2.2.1 E-survey_1_

##### 2.2.1.1 Participants

Participants comprised a purposive sample that were required to: a) be over 18 years of age; b) run for a minimum of 30 min per week prior to their most recent pregnancy; and c) have given birth within the last two years. The two-year timeframe was chosen to align with previous research (8), as well as a recent consensus statement (34) that bases this definition on recommendations for breastfeeding (35) and postpartum depression outcomes (36). Participants provided written, informed, voluntary consent prior to participation. Ethical approval was received from Cardiff Metropolitan University's Ethics Committee (project approval number: PGR-5432).

##### 2.2.1.2 Procedure

An open e-survey (e-survey_1_; see Supplementary Material) was developed based on the existing literature by a multidisciplinary research team who had expertise in sports medicine, human movement, psychology, public health and pelvic health physiotherapy. Following pilot testing with a sample of recreational, postpartum runners (*n* = 4) and minor adjustments to the wording of questions and selection of answers, the e-survey_1_ was published on a University Qualtrics account (Qualtrics, Provo, Utah). Access to the e-survey_1_ was provided to participants via a private link that was shared through social media between March 2022 and November 2022.

The e-survey_1_ was split into two parts. The first part comprised closed-ended questions, relating to demographics, details of participants’ most recent birth and any multiple pregnancies, complications experienced during or post-birth, as well as running and physical activity habits. The second section comprised three open-ended questions relating to return to running postpartum: “What helped/is helping you return to running? (This may include but is not limited to: guidance/advice, what other people did, support, structured activities, classes/formal activities).”, “What hindered/is hindering you when returning to running? (This may include but is not limited to: physical, mental or social hinderances).”, and “What else would help/have helped your return to running?”. Participants were also asked whether they would like to volunteer to participate in future research and advisory groups. There was no incentive for this, and participants were assured their response to this would not affect anything relating to e-survey_1_.

##### 2.2.1.3 Data cleaning and preparation

Of the 920 initial responses, 362 were removed due to incomplete responses, 37 were ineligible due to their responses about time since birth, three chose not to consent and exited the e-survey_1_ and fifteen were duplicate responses, resulting in a final sample of 503 participants. Duplicate responses were checked via name and date of birth, with the first entry kept in the case of a duplicate. The recruitment rate was 86.4%, while the completeness rate was 63.3%. All identifying participant data was removed from the data set, and participant responses assigned a unique ID and stored securely.

##### 2.2.1.4 Data analysis

Relevant medians with interquartile ranges (IQR), or means with standard deviations, alongside proportions (%) were calculated for the closed-ended responses to provide demographic and descriptive sample data. Time since birth (months) was calculated as the difference between the e-survey_1_ completion date and date of respondents’ most recent birth entered on the e-survey_1_. Open-ended responses regarding return to running were analysed qualitatively using thematic analysis (37). Raw text responses were organised into a word document and imported into NVivo [NVivo 12, version 12.6.1 (4859); QSR International Pty Lt, Chadstone, Australia] for iterative inductive analysis.

#### 2.2.2 Focus groups

##### 2.2.2.1 Participants

E-survey_1_ participants who consented to being contacted to take part in future research were recruited to participate in focus groups to obtain more in-depth insights. Participants were part of a purposeful, stratified sample, to reflect a broad range of country of residence to account for any geographic-specific similarities or differences (Table 1). A final sample of 17 mothers participated in the focus groups. All focus group participants had returned to running, with a median time of 10 weeks (IQR: 6–12), and 47% had returned to at least their pre-pregnancy level (*n* = 8). Participants provided their written, informed consent prior to the focus groups.

**Table 1:** E-survey_1_ respondent and focus group participant demographics.

Characteristic/demographic factor	E-survey_1_ respondents	Focus group participants
Age at time of e-survey_1_ completion median (IQR), years	35 (32–37)	33 (32–36)
Time since birth, median (IRQ), months	10 (5–16)	14 (7–18)
Years running, median (IQR)	10 (7–15)	10 (9–15)
Pre-pregnancy:		
Running mileage, median (IQR)	12 (7–20)	10 (9–15)
Runs per week, median (IQR)	3 (2–4)	3 (3–4)
During pregnancy:		
Ran, *n* (%)	353 (70%)	12 (71%)
Week stopped running, median (IQR)^a^	28 (20–34)	32 (29–38)
Did not stop running, *n* (%)	22 (6%)	0 (0%)
Running mileage, median (IQR)	2 (2–3)	2 (2–3)
Runs per week, median (IQR)	6 (4–10)	6 (5–7)
Number of children, median (IQR)	2 (1–2)	2 (1–2)
Delivery mode:^b^		
Vaginal, *n* (%)	313 (62%)	8 (47%)
Vaginal assisted, *n* (%)	65 (13%)	5 (29%)
Elective caesarean, *n* (%)	55 (11%)	1 (6%)
Emergency caesarean, *n* (%)	70 (14%)	3 (18%)
Stress urinary incontinence:		
Pre-pregnancy, *n* (%)	50 (10%)	0 (0%)
During pregnancy, *n* (%)	130 (26%)	3 (18%)
Postpartum, *n* (%)	151 (30%)	2 (12%)
Perineal tears, *n* (%)	228 (45%)	7 (41%)
Birth complications, *n* (%)	300 (60%)	11 (65%)
Postpartum complications, *n* (%)	91 (18%)	2 (12%)
Country of residence, *n* (%):^c^		
England	274 (54%)	5 (29%)
United States of America	65 (13%)	1 (6%)
Wales	58 (12%)	5 (29%)
Scotland	30 (6%)	2 (12%)
Ireland	22 (4%)	1 (6%)
Northern Ireland	20 (4%)	-
Other^d^	34 (7%)	3 (18%)
Ethnicity, *n* (%):		
White	490 (97%)	17 (100%)
Mixed/Multiple Ethnic Group	10 (2%)	-
Any other ethnic group^e^	3 (1%)	-

E-survey_1_ refers to the first e-survey used in this study. Demographics for both columns are taken and calculated from time of completion of e-survey_1_.

^a^ Week stopped running during pregnancy median, excludes 22 runners who did not stop.

^b^ Delivery mode *n* (%) is based on most recent birth prior to completion of the e-survey_1_.

^c^ Country of residence relates to country during pregnancy.

^d^ Other included Germany, Belgium, New Zealand, Austria, Norway, United Arab Emirates, Jersey, Canada, Australia and Singapore.

^e^ Any other ethnic group included Danish and Iranian (*n* = 1).

Mixed/Multiple Ethnic Group: Asian (*n* = 2), White and Asian (*n* = 4), White and Black Caribbean (*n* = 2), White and Black African (*n* = 1), White American and Mexican (*n* = 1), White British and New Zealand Māori (*n* = 1), White and Chinese (*n* = 1).

##### 2.2.2.2 Procedure

Following a pilot, three online focus groups were held via Microsoft Teams, lasting between 58 and 60 min and conducted as one group of five and two groups of six participants. The focus group sessions were conducted using a semi-structured guide based on the e-survey_1_ findings, and the themes from the open-ended e-survey were used as probes. The guide and associated probes were designed to elucidate the antecedents, determinants and consequences of return to running, as well as what participants would like to be included in a return to running intervention. The focus group guide comprised four main questions: 1) what participants regarded as a “successful return to running”; 2) what helped or was helping participants’ return to running; 3) what hindered or was hindering participants’ return to running; and 4) what a return to running intervention or programme would ideally look like. All focus groups were recorded via a Dictaphone. Multiple strategies, used in previous research, were used to minimise any potential negative effects of conducting focus groups online as opposed to face-to-face (38). For example, participants were contacted for a one-to-one test call prior to the focus group to ensure there were no technical problems, establish house rules, build rapport, and address any questions in a confidential environment. All participants were encouraged, but not obligated to use cameras, asked to remain on mute unless speaking to avoid background noise and use the raise hand function when they wanted to contribute, to avoid talking over each other. Participants were asked to ensure that they were either in a private room where others could not hear the conversation, or to use headphones, to ensure confidentiality of what was discussed. A 10-min cut-off after the start of the focus group was agreed so that participants knew that if they were late and did not join the call before this, they would be reassigned to a future group, if possible, to avoid disrupting the discussion. Finally, participants were informed that after the focus group, they could send via the email/chat function any further information; however, no participants did this.

##### 2.2.2.3 Data preparation and analysis

Focus group recordings were transcribed verbatim and analysed using inductive thematic analysis (37). This analysis followed the same process as the e-survey_1_ analysis and was conducted in NVivo [NVivo 12, version 12.6.1 (4859); QSR International Pty Lt, Chadstone, Australia].

#### 2.2.3 Parallel biomechanics study

A parallel biomechanics study was undertaken during the “Discover” phase. The aim of this exploratory study was to understand pelvic loading across different treadmill running conditions and overground exercises and develop a graded loading pathway to gradually re-load the pelvic floor. This therefore informs the ‘physical’ training programme aspect of the intervention [for more information regarding the methods and findings, see (33)]. Similarly to studies that use healthy cohorts to develop injury rehabilitation pathways, this study was not conducted with a postpartum sample. This was due to the practicalities of recruiting postpartum participants during a time where new mothers have competing priorities and also ethical considerations around the lack of prior knowledge of the pelvic loading demands of these activities. However, now that the pelvic loading during these activities is better understood via the use of wearable technology, testing with a postpartum sample in the field is a recommendation for future research.

### 2.3 Define

The second phase of the Double Diamond framework (“Define”) includes using information from the “Discover” phase to define the challenge in a different way (26). This involved identifying key aspects of what the potential intervention should involve, and how this could be developed. The key findings from the “Discover” phase that could be realistically integrated into a return to running intervention were identified, summarised, and drawn together. This provided the initial ideas used in the next phase of the co-design process, the “Develop” phase. These initial ideas could later be changed or discarded during the co-design process as well as new, related ideas being added. During this “Define” phase, a PowerPoint presentation was created for future use with an Advisory Group in the “Develop” phase and included some initial designs for what the intervention could look like based on these initial ideas from “Discover”.

### 2.4 Develop

The third phase of the Double Diamond framework (“Develop”) includes co-designing with a wide population and answering the more clearly defined problem.

#### 2.4.1 Advisory group members

Postpartum runners who consented to involvement with the Advisory Group were recruited. A purposive, stratified sampling method was employed, with the aim of working with an Advisory Group that was representative of the e-survey_1_ sample, based on a range of e-survey_1_ characteristics. The characteristics chosen to inform this purposive sample are outlined in Table 2, with a justification of why it is important to represent postpartum runners with these specific characteristics. Seven members were recruited (Table 2), who provided their written, informed and voluntary consent. Ethical approval was granted by Cardiff Metropolitan University's Ethics Committee (project ref: PGR-9362). The mean age of members who participated in the Advisory Group at the time of the first meeting was 38.6 ± 3.6 years. All participants who originally expressed interest in being involved in the Advisory Group but were not able to be incorporated into this sample, were invited to be included in later parts of the study as a wider ’sense-check group’.

**Table 2:** Advisory group member characteristics considered during purposive sampling. The targeted proportions were based on findings from e-survey_1_ and focus groups, and the characteristics of the final sample are presented.

Characteristic	Targeted proportion	Justification	Sample who participated (*n* = 7)
Delivery mode	75% vaginal (including forceps delivery)	Different rehabilitation needs based on delivery modes as evidenced by: E-survey_1_ and focus groups findings Caesarean delivery decreased odds of postpartum running-related stress urinary incontinence (8) Operative delivery e.g., caesarean or forceps have longer recovery times compared to uncomplicated vaginal birth (47)	∼86% vaginal (including forceps delivery)
	∼25% caesarean		∼14% caesarean
Perineal tears	Yes ∼ 45%	There may be additional healing time that needs to be considered based on: Childbirth-related trauma and pelvic floor dysfunction (which encompasses perineal tears) should be assessed in the review stage of 6Rs framework (10). Not a factor that affected return to running, but suggested to be due to timeframe for return to running (8). No previous guidelines consider specifics of normal deliveries which required stitches e.g., perineal tear/episiotomy (19).	Yes 43%
Postpartum complications	Yes ∼ 18%	Capturing those people who have problems that delay their return (e-survey_1_ and focus groups).	Yes 43%
Postpartum running-related stress urinary incontinence	Yes ∼ 30%	More understanding needed: Assessment of pelvic floor dysfunction in review stage of 6Rs (10). Greater odds of experiencing postpartum running-related stress urinary incontinence if have returned to running (8). Not statistically associated with not having returned to running (8), however qualitatively reported as reason for stopping/not returning to running (7).	Yes 29%
First v multiple pregnancy	First ∼ 50%	Prior experience of returning to running (e-survey_1_ and focus groups).	First 71%
		Having more than one child increased odds of returning to running to pre-pregnancy level (8).	
Returned to running?^a^	Yes ∼ 80%	Seeking different perspectives if successful or not in returning to running.	Yes 71%
Ran during pregnancy	Yes ∼ 70	Running during pregnancy increased odds of return to running postpartum (8).	Yes 57%

E-survey_1_ refers to the first e-survey used in this study.

^a^ Whether or not Advisory Group members had returned to running was based on their response (yes/no) at e-survey_1_ completion date.

#### 2.4.2 Co-design procedure

The iterative co-design process took approximately one month (June 2024). There were three online (Microsoft Teams) Advisory Group meetings, each lasting approximately one hour. The researcher presented various aspects of the proposed return to running postpartum intervention (originally developed in the “Define” phase) to the members and asked questions relating to this, as well as facilitating general discussion and feedback from members on how the proposed intervention could be improved. Meetings were audio recorded with a Dictaphone to aid note taking to inform changes made after the meetings. These recordings were not transcribed verbatim and deleted once notes were consolidated through the iterative process undertaken, and the final version of the intervention was refined.

Advisory Group members were also invited to an online, private Facebook Group, similar to previous research using social media groups (22, 39). The Facebook Group provided a platform to manage the Advisory Group and facilitated content to be discussed and developed further between online meetings. While members attended all online Advisory Group meetings that they were able to (session one: *n* = 6, session two: *n* = 3, session three, *n* = 4), further discussion of the content in the Facebook group ensured members viewed all information even if they could not attend a meeting. This blended, iterative approach of synchronous and asynchronous methods ensured there was sufficient depth of discussion during the meetings, whilst also providing a flexible alternative, which was important considering the time-constraints of being a mother [identified in many previous studies, as well as the e-survey_1_ and focus groups, as barriers of physical activity, exercise and running engagement (7, 12)]. A summary of the Facebook posts during the process is presented in the Supplementary Figure 1, similar to the format used previously (22). Throughout the process, the primary researcher also discussed the developments and subsequent amendments to the intervention with the research team.

Between Advisory Group Meetings Two and Three, an e-survey (e-survey_2)_ was sent via email to the wider ’sense-check group’ to gain further feedback on the intervention design (see Figure 1). This was to make sure that the perspectives of mothers who had not seen the intervention or been involved with its development were obtained, to ensure it made sense to others. The e-survey_2_ was open for approximately one week. Mothers were presented with the current draft of the proposed intervention and asked to give their feedback on how the intervention could be improved. Questions included in the e-survey_2_ were:

1. Would you like to do this intervention? (yes/no). Why? (open text box),
2. How could this intervention be improved? (open text box),
3. Do you have any other suggestions regarding this intervention? (open text box).

At the end of e-survey_2_ the ’sense-check’ group were also given the option of joining the Facebook group if they wished to continue the conversation. After all who opted to join and accepted the group rules were added, there were 18 members in the Facebook group, including the primary researcher and original Advisory Group members. E-survey_2_ feedback was acted upon and changes to the design were made to address what was suggested. During the third Advisory Group meeting (27.06.2024) the final intervention was then discussed with the original members (*n* = 7). Following this there was a four-week period where the Facebook group remained open to allow any final comments and refinements to be made. The research team were also consulted to proofread and suggest any other changes. When this concluded, the Facebook group was closed (02.08.2024), and the intervention was finalised.

## 3 Results

### 3.1 Discover

#### 3.1.1 Quantitative e-survey_1_ results

On completion of the e-survey_1_, 81% (*n* = 405) of the cohort had returned to running at a median time of 12 weeks (interquartile range: 8–16) to first post-birth run. Just over a third of those who had returned to running had returned to at least their pre-pregnancy level (*n* = 146) and 70% of respondents ran during their pregnancy. More information regarding respondent demographics is displayed in Table 1. Most (93%; *n* = 466) participants ran on concrete, 49% (*n* = 247) on trails, 29% (*n* = 144) on grass, 5% (*n* = 27) on other surfaces (specified as: treadmill (*n* = 17), track (*n* = 4), mix (*n* = 2) beach (*n* = 1) astroturf (*n* = 1), dirt (*n* = 1)), and 3% (*n* = 15) on sand. There were 55 participants (11%) who did not engage with any other physical activities other than running from pre-pregnancy through to postpartum. In contrast, 80%, 72% and 75% participated in other physical activities pre-pregnancy, during pregnancy during postpartum, respectively. Some examples of other activities included, but were not limited to, team-based land sports, water sports, individual sports, fitness/gym and weightlifting,

#### 3.1.2 Qualitative e-survey_1_ and focus group results

**Figure 2:**
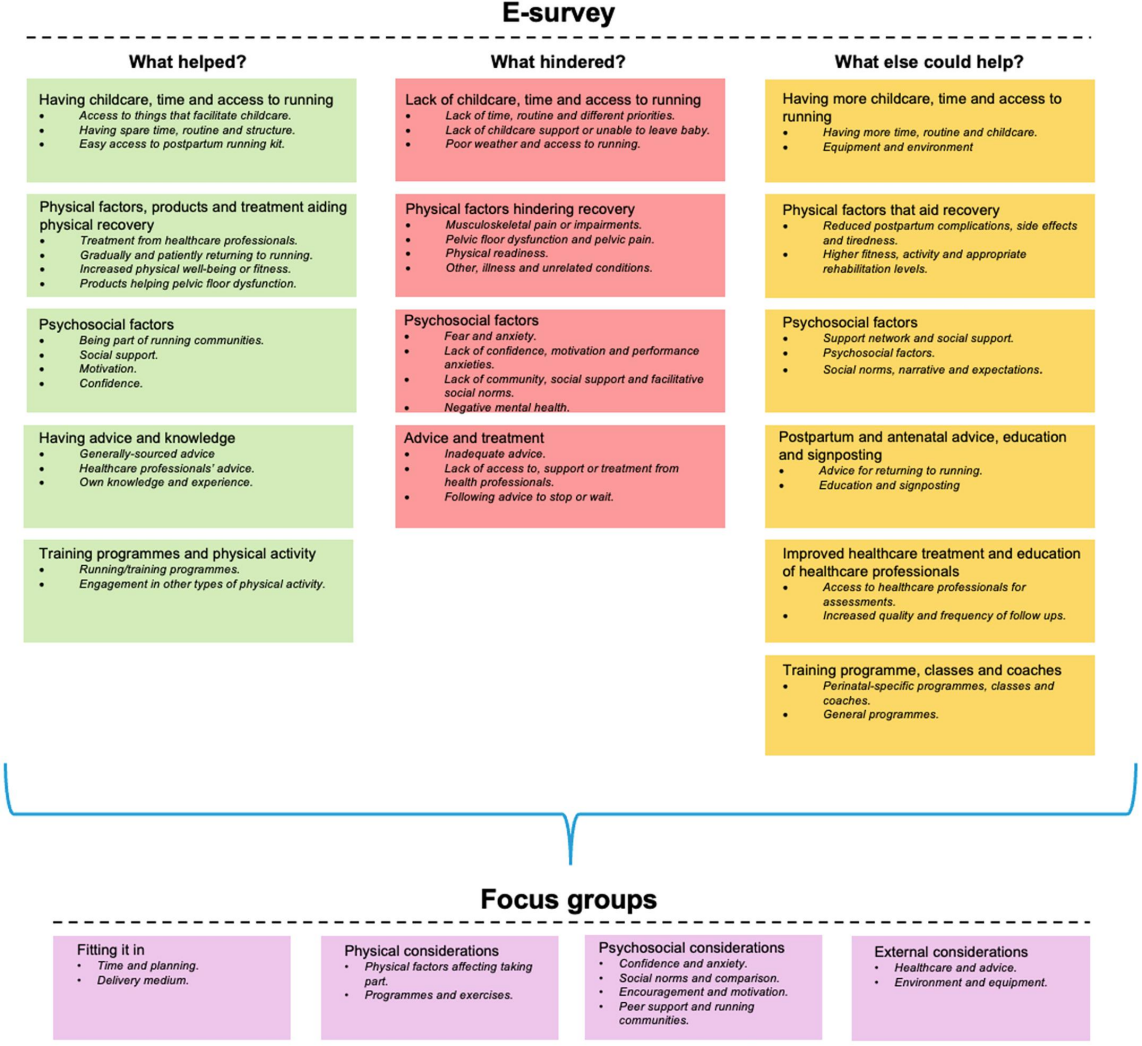
Themes and sub-themes (bullet points) developed from e-survey_1_ and focus group transcripts for what helped, hindered and what else could help return to running postpartum. While participants used the term ‘confidence’, this included a situational confidence (self-efficacy). E-survey_1_ refers to the first e-survey used in this study.

During data analysis fifteen themes were developed across the three e-survey_1_ questions, regarding what helped (five themes), hindered (four themes) and what else could help return to running postpartum (six themes; Figure 2). These findings informed the focus group discussions. During analysis of the focus group transcripts, four themes were developed including: a) fitting it in; b) physical considerations; c) psychosocial considerations; and d) external considerations (Figure 2). Examples of participant quotes for themes and sub-themes can be found in Table 3.

**Table 3:** Themes and sub-themes developed from the focus groups and example participant quotes.

Themes and sub-themes	Participant quotes
Fitting it in Time and planning Intervention delivery medium	…just trying to draw all that information that we've all spent hours and hours individually looking for, asking people, trawling the web… because when you're sleep deprived and you have a child…you're just not thinking straight and you don't have the mental load just to be sat there for hours and hours researching this, you literally just want to get out and start running again…
	…one of the things that I've struggled with is like “oh I suddenly have half an hour, I can I can go for a run right now” and I haven't planned it… and all of a sudden I'm like “oh it feels too much of a rush…I hadn't planned it I don't know where I'm running, I don't know how far I'm running …” … something that's like a *planner* [emphasis added]…
	…anything that's convenient so something like an app would be handy … you don't want to have to spend a lot of time sort of researching your … own things so like I find apps are quite easy … that's why I like the Couch to 5 K because it's an app, I could look it up, see who likes it and … [during] breastfeeding … you've got your hand free you're able to sort of have a little look on your phone…
Physical considerations Physical factors affecting taking part Programmes and exercises	…when I was pregnant with my first, you know, I was still running at 37 weeks, with my second I stopped at about 10 … I was still active … still running around after a toddler … but my body had forgotten how to run after that extra 20 weeks … my body certainly changed more after the second one…
	… I went back too soon … blew through my stitches, they had to redo my stitches … it set me back another six weeks and even at that point I was still in a lot of pain, and it was just awful.
	…I've done Couch to 5 K before and I think it's really good, it's a good way to return to running, but wouldn't it be nice to have something similar that's more designed for women who've had babies … designed for maybe an experienced runner who… has had children basically … [regarding the Couch to 5 K] it's a gradual build up … it's great for building up slowly and it helps you to avoid injury … and to get your fitness back up, I'd say that's probably the best part of it
	…maybe some exercises you can do in the period where…you have to stop for 6 weeks…almost pre-running exercises to get you in a good place, just really simple, gentle things you can do…to prepare you…
Psychosocial considerations Confidence and anxiety Social norms and comparison Encouragement and motivation Peer support and running communities	… I tried to go for a few short runs and my pelvic floor wasn't great … and it's that feeling of, “Oh my God, is this it now?” or “Will it get better [laughing]?” … “Will I be able to run without weeing myself?” … I think I was probably trying to hold my pelvic floor, or … constantly thinking about it … I think it just affects the way that you run and you move …
	… this whole body changing thing as well…none of my running stuff fits me anymore, because I was really skinny before I had the twins which was really annoying because I'm not now … and there's a little bit of … this social anxiety … knowing that I look different and everything's a wee bit more wibbly wobbly because the skin is very loose and stuff … it's just putting me off going out a lot, especially because it's getting warmer, so I wouldn't want to wear a big baggy top to cover everything … but I don't want to wear a tight vest top where everyone can see everything moving … it's a new experience for me because I have always been quite slim … so I'd always try to run somewhere where I know people aren't going to see me and … I know in the back of my head it's a really stupid thing to stop me going out but it's there …
	… hopefully this can sort of lead on to a shift in people's thinking about exercising when pregnant as well because … I felt judged sometimes, the fact that I was still running and still exercising whilst pregnant for both my pregnancies. I often got told “you shouldn't be doing that, it's dangerous for the baby” … even though I knew in my heart of hearts I was absolutely fine … I think it's sort of ingrained in some people that the moment … you get pregnant, you should stop all sorts of exercise … it's almost frowned upon …
	… I've got one friend … she's got two children, but she just managed to sort of bounce back and regain her fitness very quickly. We had children around the same time, and I felt that I couldn't keep up with her so that actually [laughing] made me feel worse to be honest…
	… some motivational things maybe from other mums that have got back into it [running] to say, “Don't worry if it feels really overwhelming at the moment … just take it easy, even if you go out for 10 min … it will come back but just be patient with it and you might feel completely weird in your body and that's normal” … just things like that so there's that … support there … and reassurance; don't just give up at the first hurdle.
	… parkrun has been a massive part of my return and like the whole community thing … just walking around with the pram at around three weeks [postpartum] and it's just felt really good to be part of the community …
External considerations Healthcare and advice Environment and equipment	…I agree with what's been said about the initial assessment … there needs to be some consideration of when did you stop running … what has your body been used to and what sort of symptoms did you have during pregnancy and, what type of birth did you have … this global physical assessment including your pelvic floor but more broadly of where are you now and what is going to be safe and how … quickly can you … progress … just within the six of us [participants] there's been such … varied experiences with birth and our ability to exercise…
	…the level of people who hold themselves out to be experts, varies quite significantly … I certainly went to some exercise classes where they … tailored every exercise knowing the type of birth that you had … how far along in recovery you were, how far you weren't, is this your first exercise class … and I went to others where they didn't even ask a single question … but these were people holding themselves out as … experts in postnatal exercise…
	…I have a lot of friends in the US and in the UK and I feel … something that Belgium has really nailed is postpartum care for women … every woman gets nine PT sessions for free after giving birth so that they can restore their pelvic floor … and that is done with a pelvic floor, specialised PT … even if you don't want to [laughing], the very first day after you give birth you are still in the hospital, there is a specialist…
	Opposite to Jennifer, I think my environment's super conducive to me wanting to run so I live in a really beautiful running area along the river … I'd say at least 10 and a half months of the year can run outside without too much struggle … so for me the motivation *is* [emphasis added] wanting to be outside.

### 3.2 Define

**Figure 3:**
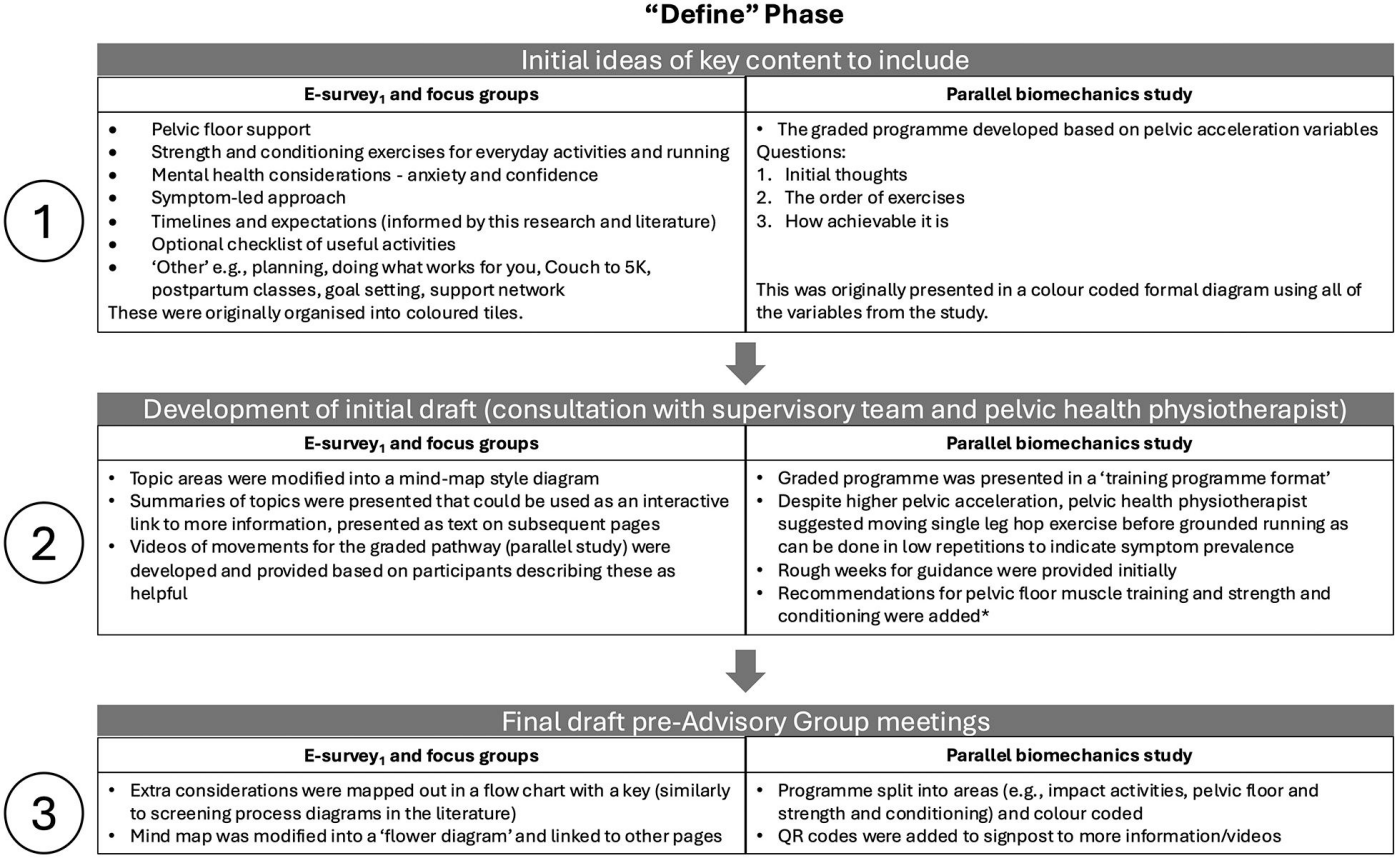
Concepts taken from e-survey_1_ and focus groups, as well as a parallel biomechanics study (33) to “define” the problem and potential solutions. These informed the initial draft of the intervention. Stages 1, 2 and 3 show how this content was modified following consultation with the supervisory team, prior to the first Advisory Group meeting in the “Develop” phase of the Double Diamond framework (26). *Based on the literature and consultation with the pelvic health physiotherapist.

Through the peer-review iterative process PowerPoint slides were modified and are evidenced in Supplementary Figure 2. Key changes made across slides included simplifications of the aims, contextualising the work in line with the UK Chief Medical Officers’ Physical Activity Guidelines (40), improving the programme design, as well as adding activity videos and additional slides. Version 3 of the slides (referenced in Figure 3 and Supplementary Figure 2) was the final draft that was presented in the first Advisory Group meeting.

### 3.3 Develop

During the co-design of the intervention, key considerations highlighted by the postpartum runners included the need for any intervention to be realistic and achievable, practical to fit into everyday life, and provide the right balance between enough information without being overwhelming. Additionally, the consensus was that the intervention needed to be user-friendly and designed in a way that appealed to “mums” and not “athletes”. Advisory Group members also suggested that endorsement or association with an official organisation might add to the credibility and trustworthiness of the guide. Following the last Advisory Group meeting, endorsement was therefore sought from Pelvic Obstetric and Gynaecologic Physiotherapy (POGP). Officially recognised as a UK-based Professional Network of the Chartered Society of Physiotherapy, POGP is a registered charitable body that aims to provide evidence-informed care and advice for the public and its members (Pelvic Obstetric Gynaecological Physiotherapy, 2020). This is achieved through POGP's social media engagement, patient and clinician areas on their website, as well as other useful resources and links that were signposted to within the guide. A summary of the feedback and key changes to the intervention are provided in Table 4.

**Table 4:** Summarised feedback during co-design of the return to running postpartum intervention.

Feedback source	Feedback
Advisory Group Meeting 1	Progressions between stages needed to be clearer and use of timelines altered Suggestions around design, including linearity and rigidity of programme needed reducing Additional exercises and guidance for exercises suggested, as well as potentially removing more complicated and unfamiliar exercises and terminology Programme looked overwhelming, would be helped by including examples of how to integrate into everyday life Guidance for those who may not have run before needed Information on how to recognise a ‘good response’ to exercise needed
Advisory Group Meeting 2	Changes made following Advisory Group Meeting 1 were well received Need for logos to add to trustworthiness of document Need to speak about mental health more broadly in the document to ensure nobody's experience is not acknowledged Add in some extra detail on pelvic floor dysfunction symptoms and where you can go (general practitioner/midwife) if pelvic health physiotherapist is unavailable More signposting to resources needed within the guide, without too many links that would make it overwhelming Some design suggestions including toning certain images down Increase clarity around example used to demonstrate progression across areas
E-survey_2_	More clarity needed on navigating the document and need to streamline information and make less overwhelming Comments made regarding design and presentation order of some information Specific comments around additional content that respondents wanted (e.g., mastitis, buggy fitness groups, strength training, relaxing pelvic floor muscles) Problems with timelines if not aligning with one's experience Ensuring that the document was widely available and could be accompanied by support
Facebook Group	Some indication on frequencies of activities wanted Simplify and reduce links to most relevant information Majority happy with presentation of ‘stages’ and ‘areas’ of programme as they are Clarity around repeats and rests on video voiceovers requested Consensus gathered on any preferences for terminology (e.g., members did not mind whether ‘exercise’ or ‘physical activity’ was used throughout the guide)
Advisory Group Meeting 3	Front cover and name of document discussed Good reaction to the updated navigation of the document Ways of including frequency of activities discussed and idea of planner with how you can fit physical activity into your week to make up ∼150 min added Moved single leg hop exercise to Stage 2 as seen as could be a barrier to progress from Stage 1. Also renamed single leg ‘low’ hops to stress it isn’t a highly demanding activity Presentation of information regarding delivery mode tweaked to incorporate unique birth experience as a whole, and for acknowledge some mothers may have been through nearly a complete vaginal and caesarean birth with emergency caesarean Minor design changes and format of delivery discussed—PDF preferred, and suggestion for future potential mobile app development

E-survey_2_ is the second e-survey used in the study.

**Figure 4:**
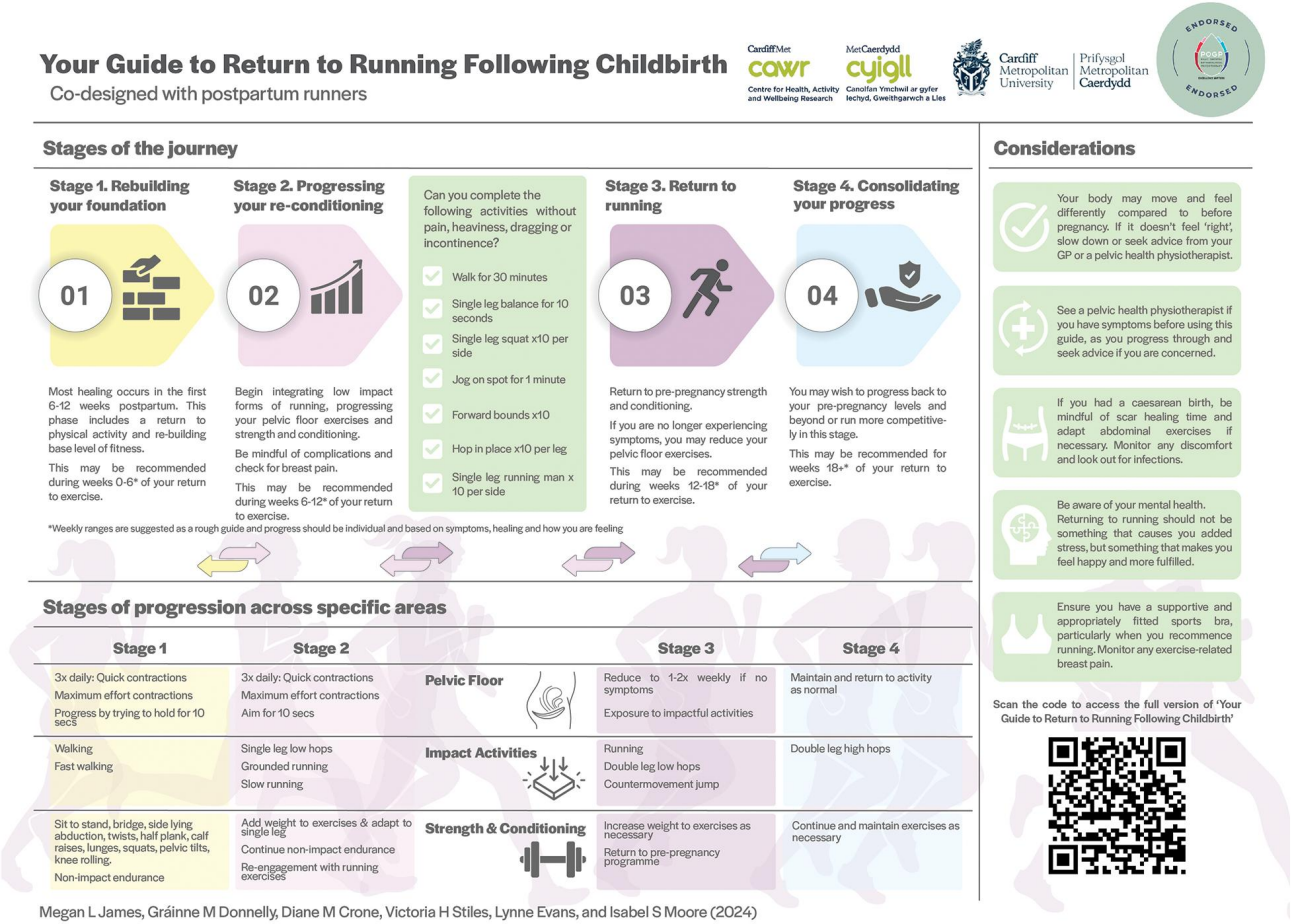
Summary infographic of the co-designed guide for return to running following childbirth. The QR code links to the full guide. This guide has not yet been tested.

As a result of the “Develop” stage, an intervention accounting for biopsychosocial factors and lived experience, was produced in the form of a guide. The final document was required to be stored on Figshare (Cardiff Metropolitan University's repository; link: https://doi.org/10.25401/cardiffmet.26403592.v1), to enable open, public access. Furthermore, additional information for opening the guide's Supplementary Files and guide itself were included on the ‘how to’ page for specific reading of the resources through Figshare. Subsequently, a summary infographic was produced for publication and dissemination purposes, which the members agreed would be useful in Advisory Group Meeting 3. The full guide can be accessed through the QR code on the infographic (Figure 4), which has since been officially endorsed and published in POGP's booklet [ (41), https://thepogp.co.uk/_userfiles/pages/files/resources/241419pogpfffuture_1.pdf], aiding dissemination further.

## 4 Discussion

The aims of this study were to: 1) investigate the barriers to and facilitators of postpartum return to running; and 2) co-design a postpartum return to running intervention that is underpinned by clinical recommendations. This process was guided by the Double Diamond framework (26). Four themes relating to what needed to be considered in a return to running intervention were developed. These were: a) fitting it in; b) physical considerations; c) psychosocial considerations; and d) external considerations (Figure 2). Following Advisory Group meetings, discussion via a Facebook Group and an e-survey_2_, a return to running intervention was designed in the form of a guide. The guide accounts for biopsychosocial factors in a way that many postpartum physical activity frameworks to date have failed to do. The Advisory Group highlighted the need for the guide to be realistic, achievable, and practical, providing the right balance of information. Finally, the guide needed to be user-friendly and designed in a way that appealed to and reflected being a “mum” rather than an “athlete”. Each phase of the Double Diamond process is discussed below with regards to reflections on the process and previous literature.

### 4.1 Discover

To address the shortcomings of previous research that has not investigated running specifically (11–13), identified facilitators, nor adopted a user-centred approach (7, 8, 18), the e-survey_1_ and focus groups conducted in the present study investigated both the barriers and facilitators of return to running postpartum (10). Building on previous research (7, 8, 18), the e-survey_1_ and focus groups revealed that many mothers had changed their expectations to run more recreationally and enjoy a symptom-free experience, whereas others wanted to return to competitive races and pre-pregnancy levels of running. Time spent researching how to return to running contributed to mothers’ lack of time for running and that more help was needed with conditioning for everyday activities. Participants also highlighted the need for psychological screening, for example awareness of exercise addiction, demonstrating the importance of biopsychosocial factors. Additionally, participants explained that there were many differences in healthcare between countries, which needs to be standardised. Finally, postpartum runners outlined a desire for a specific, graded programme, a recommendation that has previously been made by academics and clinicians (10, 19, 42, 43), but not by postpartum runners.

### 4.2 Define

As return to running postpartum is a relatively unexplored area, it was deemed critical to involve a distinct “Define” phase, prior to delving into the “Develop” phase. A broad range of perspectives to aid in this iterative peer-review process was fostered by having a multidisciplinary research team. This was particularly important as information gathered in the “Discover” phase spanned a broad range of disciplines and needed to blend together in the design of a potential biopsychosocial intervention (10, 44). Further, as well as the end-users in the Advisory Group, several members of the research team had lived experience of return to running postpartum, and this provided useful insights as well as aiding sense-checking. This phase therefore facilitated the integration of factors gathered from a range of studies, methods and perspectives, to ensure employment of an *inter* rather than *multi*disciplinary approach, prior to the “Develop” phase.

### 4.3 Develop

The co-designed guide addresses the need for a person-centred, interdisciplinary, biopsychosocial approach, previously recommended for return to running postpartum (10, 44). As such it reflects a novel, holistic approach that embraces physical, social and mental imperatives that are essential considerations for the women using it to support their return to running. For example, the physical aspect of the programme provides pre-conditioning exercises prior to running in stages one and two, as suggested previously (43). Some of these low impact and strength and conditioning activities could help mothers meet the demands of daily activities, highlighted as important in the focus groups. Furthermore, guidance and signposting to other reputable resources were included in one resource, to reduce the time that postpartum runners spend searching for information. It was also evident that mothers needed a symptom-led approach, and while approximate timeframes were useful as a guide, criteria-based (e.g., presence of symptoms) as opposed to time-based progression was preferred. While this is in line with existing clinical advice for postpartum return to running (19, 42, 43), confirmation that postpartum runners approve of this approach is important to note. Specifically, it indicates that postpartum runners believe they can adopt this self-led strategy and manage their progression and regression between stages. Until further objective criteria, similar to those used in sports injury rehabilitation [e.g., (45)], are established for postpartum progression, a symptom-led approach provides a useful alternative, serves as a roadmap for future research and helps individualise the use of the guide.

### 4.4 Strengths

This novel mixed-methods study has several strengths. For example, the combination of the e-survey_1_ and focus groups allowed a breadth and depth of investigation of experiences of return to running postpartum, addressing some of the limitations of previous research. Also, online focus groups allowed greater representative access to and geographical spread of participants than face-to-face discussions, especially in a time-restricted population. Further, the iterative design and various strategies employed yielded a large amount of stakeholder feedback that informed the potential intervention design. It should be noted that there was varying attendance at the online sessions, perhaps indicative of demands on mothers’ free time. As a result, use of asynchronous and synchronous approaches, through online meetings and inter-meeting Facebook discussion may provide a useful approach for co-design with other hard-to-reach populations. There were no requirements to join on a particular device, and many mothers joined the meetings on their mobile phone which increased accessibility further. The meetings were held in the early afternoon (one at 12:45pm and two at 1:30pm), which emerged as a suitable arrangement for this population. Finally, the strength of the interdisciplinary and co-design approach was highlighted when Advisory Group members suggested moving certain exercises from the end of one stage to the start of the next, to make that initial stage seem more achievable. They explained that this would be less de-motivating for users who would otherwise not be able to progress beyond that stage if they could not perform that exercise without symptoms (Table 4). This interdisciplinary, co-design approach therefore ensured an accessible and feasible approach that was not constrained by any one disciplinary approach to return to running.

### 4.5 Limitations and future directions

As with any research, this study is a not without some limitations. Specifically, participants may be prone to recall bias, particularly for those further into the postpartum period. It is also possible that there could have been sampling bias, with mothers who experienced a more difficult return to running more likely to participate than those who had an easier return. This however means that while the barriers identified in the e-survey_1_ and focus group are those that require most urgent attention, there may be some facilitators that are yet to be uncovered. Additionally, not all Advisory Group members were able to attend all meetings and there were some disagreements between members. Therefore, it is acknowledged that decisions were made that not all may have agreed with, and therefore this intervention may not be generalisable to all postpartum runners. As with all recommendations and guidelines developed using co-design, this guide cannot be assumed to be a solution for all end-users. However, it has been designed in line with recommendations from clinical practice, the available research evidence, and uniquely, co-designed with postpartum runners, through an iterative multi-method design. This robust approach provided a guide that is flexible enough that users can adapt it to their needs and seek additional support if needed. Future research should test the feasibility and effectiveness of this guide in postpartum runners. Subsequently, this guidemay be further tailored for runners of different levels and experiences, as well as any other sub-groups that may require more targeted return to running advice.

## 5 Conclusion

This mixed-methods, co-design study provides an increased understanding of barriers to and facilitators of return to running postpartum and culminates in, to the authors’ knowledge, the first co-designed guide for return to running postpartum. This guide is based on the existing research literature, evidence from the multiple phases in this study, clinical recommendations and collaboration with postpartum runners. It also accounts for the multiplicity of biopsychosocial factors that many postpartum physical activity frameworks have seemingly overlooked. As a result, it has provided for a rigorously designed and clinically endorsed resource. It is hoped that future use of this guide will act as a driver for increased re-engagement with running postpartum, while facilitating healthy lifestyles for mothers and their children.

## Data Availability

The raw data supporting the conclusions of this article will be made available by the authors, without undue reservation.
