# Supplementary material for: An interdisciplinary, co-designed guide for return to running postpartum—a mixed-methods study
Source: Front Sports Act Living. 2026 Mar 30;8:1771882. doi: 10.3389/fspor.2026.1771882 (PMC13070941; doi:10.3389/fspor.2026.1771882)
Supplement: Supplementary file 1 [file Datasheet1.pdf]

E-survey[1] exploring barriers and facilitators of return to running postpartum

The e-survey had 30 items 15 pages, 1-7 items per page. Due to the need to appear in a logical order, the questions were not randomised and adaptive questioning ensured that participants were only asked questions that were applicable to them, based on their previous responses. Participation was voluntary and there were no incentives. All questions forced a response to ensure completeness, and a back button allowed participants to review and if necessary, change answers. IP address tracking was turned off.

<u>Return to running after giving birth e-survey (R2RBS)</u>

<u>Part 1 - Questions about you, your childbirth history, running and activities</u>

This first section will ask for information about you, your (most recent) birth and the activities and running that you have engaged with before, during and after pregnancy.

Yourself and childbirth history

1. What is your full name? (Text box)
2. What is your date of birth? (Text box)
3. What is your country of residence? (Drop down options)
4. What is your ethnic group? (Drop down options)
5. When did you give birth (please provide the date of your most recent birth)? (Text box)
6. Where did you reside for the majority of your most recent pregnancy? (Drop down options)
7. How many weeks pregnant were you when you gave birth (for your most recent birth)? (Drop down options)
8. How many children have you given birth to (including your most recent birth)? (Drop down options)
9. Was your most recent birth a multiple pregnancy? Yes/no. If yes, please specify how many children you gave birth to during your most recent pregnancy (Text box)
10. Were any of your previous births a multiple pregnancy? Yes/no/N/A- I have only had one birth. If yes, please provide details of the year of this pregnancy and how many children you gave birth to during your multiple pregnancy. (Text box)
11. What was your delivery mode during your most recent birth? (Options: vaginal, vaginal assisted, caesarean)
12. (If answered caesarean) Was your caesarean in your most recent birth elective or emergency? (Tick box for each)
13. Did you experience stress urinary incontinence before, during or after your most recent pregnancy? (Tick box for each, and “no, not during any of these periods”.)
14. Did you experience any birth complications during your most recent birth? Select all that apply.
    - Perineal tear (if yes, please specify the degree of tear in the box below. 1st - skin graze, 2nd - muscle tissue in perineum, 3rd - muscle tissue in perineum and external anal sphincter, or 4th - extends through entire anal sphincter) ☐
    - Cardiovascular complications (e.g., high blood pressure, heart complications ☐)
    - Excessive bleeding (haemorrhage) ☐
    - Other (free text) ☐
    - None ☐
15. Were there any medical or post-birth complications associated with your most recent birth? Select all that apply.
    - Wound infection ☐

- Post-birth psychosis
- Sepsis
- Other (free text)
- None

Running and activities

16. How many years have you been running? (Drop down)
17. On average, how many runs did you do per week before your most recent pregnancy? (Drop down)
18. On average, how many miles did you run per week before your most recent pregnancy? (Drop down)
19. Did you run during your most recent pregnancy? (Yes/no)
20. (If yes) On average how many runs per week did you do during your most recent pregnancy? (Drop down)
21. (If yes) On average how many miles per week did you run during your most recent pregnancy? (Drop down)
22. Which week of your pregnancy did you stop running? (Drop down options)
23. Have you returned to running since your most recent birth? (Yes/no)
24. (If answered yes to returned to running) How many weeks after you gave birth did you return to running? (Drop down options)
25. (If yes to RTR) Have you returned to the same or a greater level of running since your most recent birth compared to before this pregnancy? (Yes/no, I run less than before my most recent pregnancy)
26. What surface(s) do you normally run on? Select all that apply.
    - Concrete/road
    - Grass
    - Trail
    - Sand
    - Other (please specify) (Text box)
27. Have you participated in any other physical activity or sport before, during or after your most recent pregnancy? Select all that apply. If yes, please provide details.
    - Before pregnancy (Text box)
    - During pregnancy (Text box)
    - After pregnancy (Text box)
    - No

<u>Part 2 – Your experiences and thoughts on returning to running/jogging</u>

Thank you for your responses so far. We are now interested to hear about your return to running experiences after giving birth. We know that the return to running journey can be very challenging, where individuals have different experiences. Therefore, we are really interested in the challenges and barriers, as well as the things that can help/facilitate return to running. We would be very grateful for as much detail as possible. Please proceed to the next page.

1. What helped/is helping you return to running? (This may include but is not limited to: guidance/advice, what other people did, support, structured activities, classes/formal activities).

2. What hindered/is hindering you when returning to running? (This may include but is not limited to: physical, mental or social hinderances).

---

3. What else would help/have helped your return to running?

To further understand the experiences and challenges of returning to running, and your specific needs and preferences, we will be undertaking future research in this area. If you are interested in participating in this (there is no obligation to participate, we will just send you information and an invitation which you can ignore or decline), please tick the following statement and fill in your email address in the box below. If you would not like to participate, this will not influence any data you have provided so far, please leave the box unticked and proceed to the following page to complete the e-survey.

I consent to being invited to participate in future research concerning this topic. ☐

As part of the PhD project mentioned at the beginning of this e-survey, we would also be grateful for volunteers to take part in an advisory group that will inform future project work. If you would like to be invited to join an advisory group (you can later decline), please tick the box, and fill in your email address. If you would not like to participate, this will not influence any data you have provided so far, please leave the box unticked and proceed to the following page to complete the e-survey.

I consent to being invited to join an advisory group. ☐

Thank you very much for taking the time to fill in our e-survey.
If you would like to know more about this e-survey or our research, please contact the research student Megan James: st20154529@outlook.cardiffmet.ac.uk. If you have queries that you would prefer to discuss with the research supervisor, please email Prof Lynne Evans: LEvans@cardiffmet.ac.uk.

END OF E-SURVEY
