# Supplementary material for: An interdisciplinary, co-designed guide for return to running postpartum—a mixed-methods study
Source: Front Sports Act Living. 2026 Mar 30;8:1771882. doi: 10.3389/fspor.2026.1771882 (PMC13070941; doi:10.3389/fspor.2026.1771882)
Supplement: Supplementary file 2 [file Image1.pdf]

Formation of Advisory Group and Facebook Group

• 03.06.24: Introduction: purpose of the Facebook group/project explained, and members directed to the group description, rules, privacy settings. Members reminded about upcoming sessions and dates.	• Text, 1 like, 0 comments
• 05.06.24: Members reminded about the session on 06.06.24. Teams link added, as well as sent via email.	• Text. 3 likes, 0 comments

Advisory Group Meeting 1 (half of initial intervention design presented to members)

• 06.06.24: Thank you email for participating in the session. Reminder that summary points to be sent over the next day.	• Text, 4 likes, 0 comments
• 07.06.24: Key points from yesterday's Advisory Group meeting summarised and any initial reflections asked for.	• Text with image, 2 likes, 2 comments
• 09.06.24: Links for resources associated with the slides attached to get feedback on.	• Text, 0 likes, 4 comments
• 10.06.24: Members reminded about the time of session on 13.06.24.	• Text, 3 likes, 0 comments
• 12.06.24: Teams link added here, as well as sent via email. Members asked to think of anything additional that they would like to discuss.	• Text, 0 likes, 1 comment

Advisory Group Meeting 2 (second half of intervention and any updated from Advisory Group 1 presented and discussed)

• 13.06.24: Key points from today's Advisory Group meeting summarised and any initial reflections asked for.	• Text with image, 4 likes, 2 comments
• 14.06.24: Discussion around updated slides from Meeting 1 for those not in Meeting 2, and changes since Meeting 2 (these changes were in the updated version presented in the e-survey).	• Text with image, 2 likes, 7 comments
• 17.06.24: Poll for wanting frequencies for activities in the guidance or not (it was presented without frequencies to begin with).	• Text with poll, 0 likes, 2 comments, 4 votes[1]
• 18.06.24: Post with new slides discussed in Meeting 2.	• Text with image, 1 like, 2 comments

Sense-check Group Complete E-survey (additional members added to Facebook Group)

• 18.06.24: Welcome post for the sense-check group who were added in as members and explanation for existing members (survey released 18th June).	• Text, 1 like, 0 comments
• 21.06.24: Links to movement/exercise videos with voice overs and sub-titles posted for feedback.	• Text with link, 0 likes, 2 comments
• 25.06.24: Information tabs that ink to flower diagram posted for feedback.	• Text with images, 3 likes, 1 comment

Advisory Group Meeting 3 (updated intervention presented, discussed and finalised)

• 01.07.24: Poll asking if members preferred 'exercise' v 'physical activity' to be used throughout the guide.	• Text with poll, 0 likes, 0 comments, 8 votes[2]
• 01.07.24: Poll asking if the document should have each stage presented one at a time (for all areas) or areas presented one at a time (with all stages).	• Text with poll, 0 likes, 3 comments, 6 votes[3]
• 03.07.24: Name check of the guide.	• Text, 5 likes, 0 comments
• 04.07.24: Final version of guide posted out asking for final feedback.	• Text with link, 1 like, 3 comments
• 10.07.24: Reminder about feedback.	• Text with link, 0 likes, 0 comments
• 11.07.24: Extended time for feedback.	• Text with link, 1 like, 0 comments
• 26.07.24: Final reminder for feedback.	• Text with link, 0 likes, 0 comments
• 31.07.24: Post detailing final changes and thanks to members (waited until seen by everyone and then closed).	• Text with image, 4 likes, 2 comments

Supplementary Figure 1. The co-design process (grey boxes) followed by what was posted on the Facebook group between these events. Note: Date of post is presented (dd.mm.yy). In the right column, information is given for type of post, likes / comments / votes (if on posts with polls). [1]All votes wanted more information on frequencies. [2]All votes did not mind which term was used. [3]4 votes preferred one area at a time with all stages.
