# Supplementary material for: An interdisciplinary, co-designed guide for return to running postpartum—a mixed-methods study
Source: Front Sports Act Living. 2026 Mar 30;8:1771882. doi: 10.3389/fspor.2026.1771882 (PMC13070941; doi:10.3389/fspor.2026.1771882)
Supplement: Supplementary file 3 [file Image2.pdf]

1	2	3

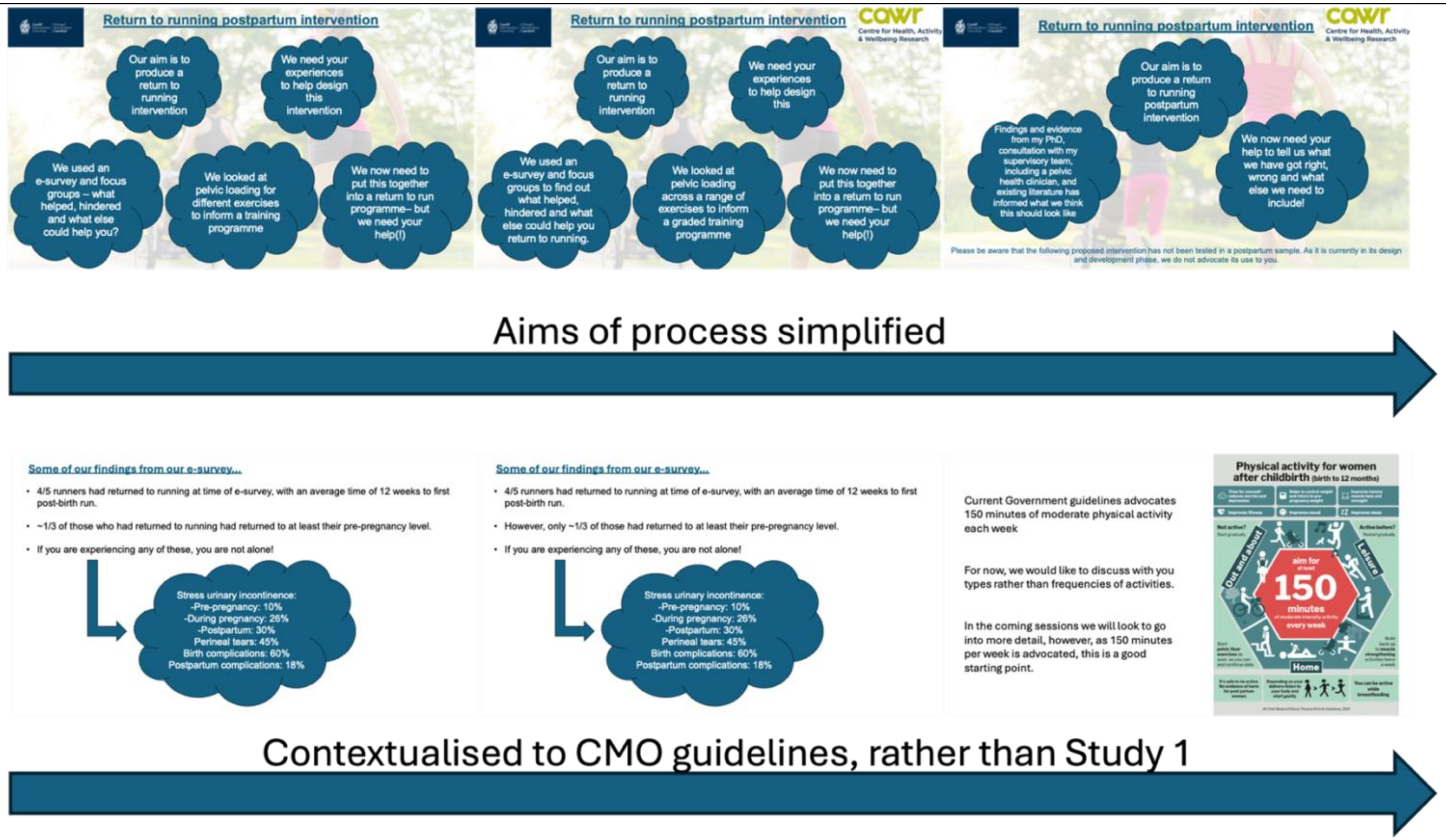
Return to running postpartum intervention
Our aim is to produce a return to running intervention
We need your experiences to help design this intervention
We used an e-survey and focus groups – what helped, hindered and what else could help you?
We looked at pelvic loading for different exercises to inform a training programme
We now need to put this together into a return to run programme– but we need your help(!)
Return to running postpartum intervention
cawr
Centre for Health, Activity & Wellbeing Research
Our aim is to produce a return to running intervention
We need your experiences to help design this
We used an e-survey and focus groups to find out what helped, hindered and what else could help you return to running.
We looked at pelvic loading across a range of exercises to inform a graded training programme
We now need to put this together into a return to run programme– but we need your help(!)
Return to running postpartum intervention
cawr
Centre for Health, Activity & Wellbeing Research
Our aim is to produce a return to running postpartum intervention
Findings and evidence from my PhD, consultation with my supervisory team, including a pelvic health clinician, and existing literature has informed what we think this should look like
We now need your help to tell us what we have got right, wrong and what else we need to include!
Please be aware that the following proposed intervention has not been tested in a postpartum sample. As it is currently in its design and development phase, we do not advocate its use to you.
Aims of process simplified
Some of our findings from our e-survey...
4/5 runners had returned to running at time of e-survey, with an average time of 12 weeks to first post-birth run.
~1/3 of those who had returned to running had returned to at least their pre-pregnancy level.
If you are experiencing any of these, you are not alone!
Stress urinary incontinence:
-Pre-pregnancy: 10%
-During pregnancy: 26%
-Postpartum: 30%
Perineal tears: 45%
Birth complications: 60%
Postpartum complications: 18%
Some of our findings from our e-survey...
4/5 runners had returned to running at time of e-survey, with an average time of 12 weeks to first post-birth run.
However, only ~1/3 of those had returned to at least their pre-pregnancy level.
If you are experiencing any of these, you are not alone!
Stress urinary incontinence:
-Pre-pregnancy: 10%
-During pregnancy: 26%
-Postpartum: 30%
Perineal tears: 45%
Birth complications: 60%
Postpartum complications: 18%
Current Government guidelines advocates 150 minutes of moderate physical activity each week
For now, we would like to discuss with you types rather than frequencies of activities.
In the coming sessions we will look to go into more detail, however, as 150 minutes per week is advocated, this is a good starting point.
Physical activity for women after childbirth (birth to 12 months)
aim for
150
minutes
every week
Out and about
Leisure
Home
Contextualised to CMO guidelines, rather than Study 1

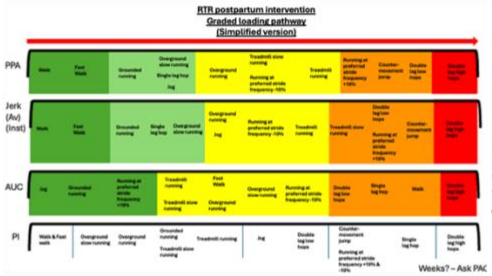

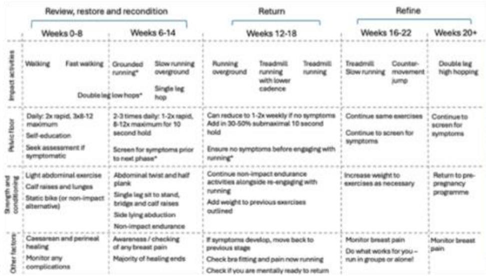

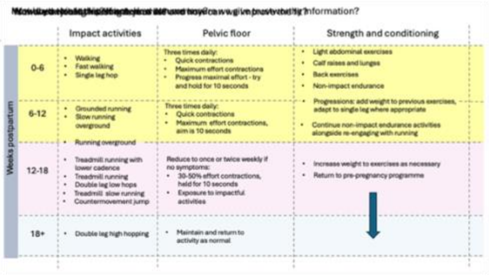

Improved 'programme' design

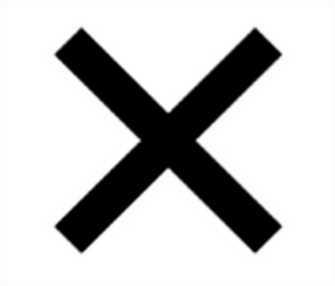

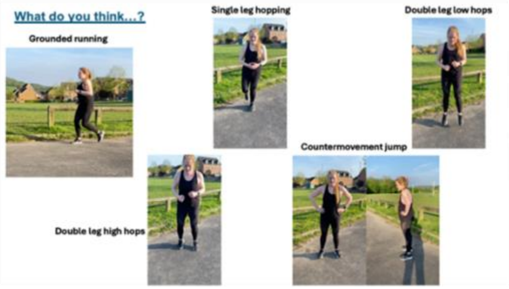

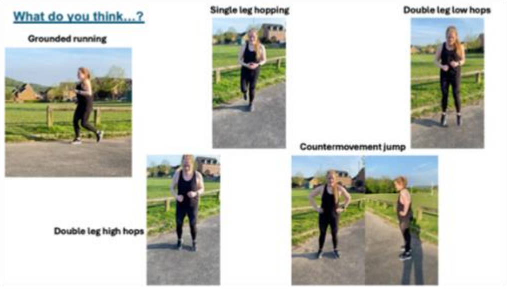

Addition of impact activity videos

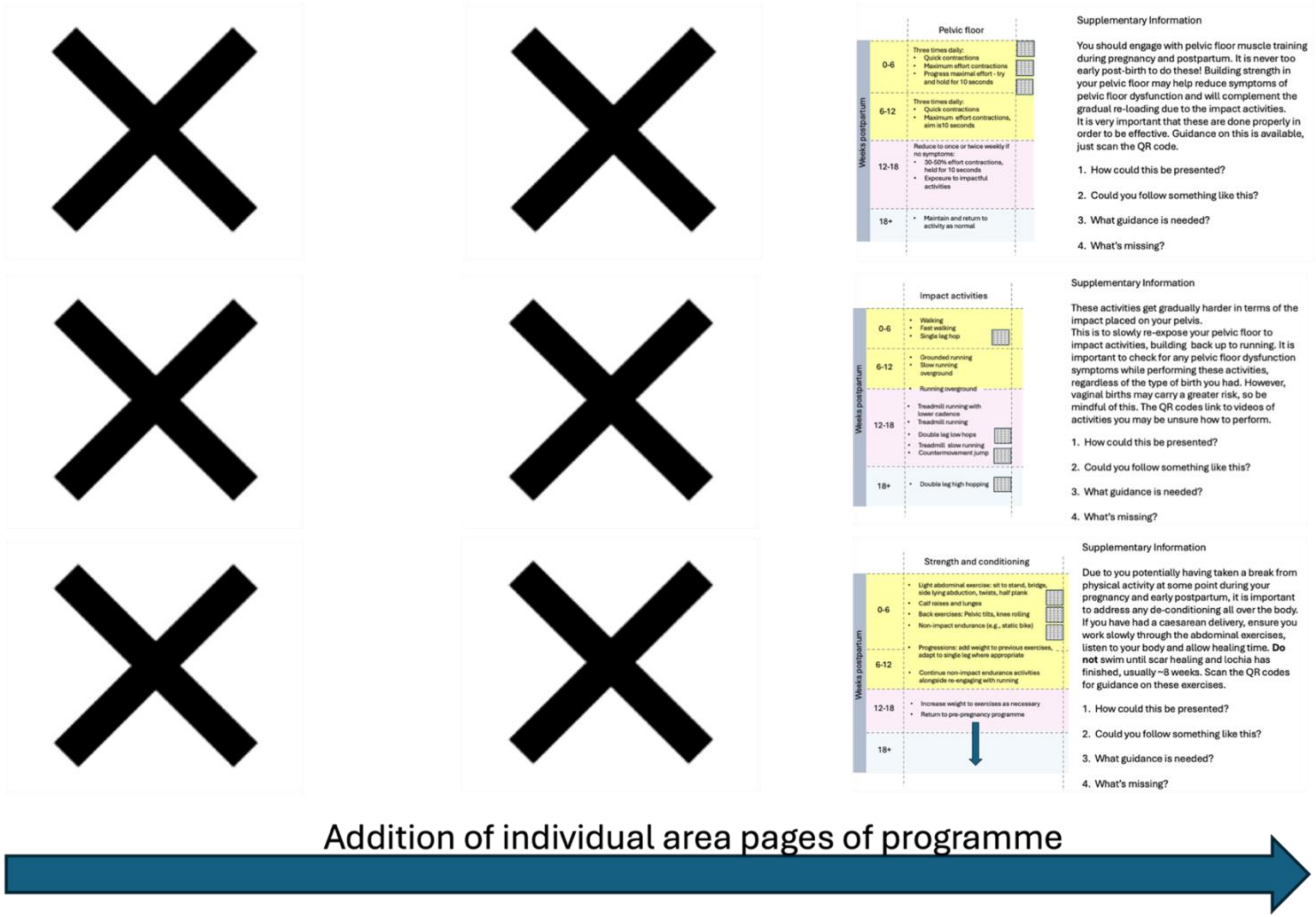
Pelvic floor
Weeks postpartum
0-6
Three times daily:
Quick contractions
Maximum effort contractions
Progress maximal effort - try and hold for 10 seconds
6-12
Three times daily:
Quick contractions
Maximum effort contractions, aim is10 seconds
12-18
Reduce to once or twice weekly if no symptoms:
30-50% effort contractions, held for 10 seconds
Exposure to impactful activities
18+
Maintain and return to activity as normal
Supplementary Information
You should engage with pelvic floor muscle training during pregnancy and postpartum. It is never too early post-birth to do these! Building strength in your pelvic floor may help reduce symptoms of pelvic floor dysfunction and will complement the gradual re-loading due to the impact activities.
It is very important that these are done properly in order to be effective. Guidance on this is available, just scan the QR code.
1. How could this be presented?
2. Could you follow something like this?
3. What guidance is needed?
4. What's missing?
Impact activities
Weeks postpartum
0-6
Walking
Fast walking
Single leg hop
6-12
Grounded running
Slow running overground
Running overground
12-18
Treadmill running with lower cadence
Treadmill running
Double leg low hops
Treadmill slow running
Countermovement jump
18+
Double leg high hopping
Supplementary Information
These activities get gradually harder in terms of the impact placed on your pelvis.
This is to slowly re-expose your pelvic floor to impact activities, building back up to running. It is important to check for any pelvic floor dysfunction symptoms while performing these activities, regardless of the type of birth you had. However, vaginal births may carry a greater risk, so be mindful of this. The QR codes link to videos of activities you may be unsure how to perform.
1. How could this be presented?
2. Could you follow something like this?
3. What guidance is needed?
4. What's missing?
Strength and conditioning
Weeks postpartum
0-6
Light abdominal exercise: sit to stand, bridge, side lying abduction, twists, half plank
Calf raises and lunges
Back exercises: Pelvic tilts, knee rolling
Non-impact endurance (e.g., static bike)
6-12
Progressions: add weight to previous exercises, adapt to single leg where appropriate
Continue non-impact endurance activities alongside re-engaging with running
12-18
Increase weight to exercises as necessary
Return to pre-pregnancy programme
18+
Supplementary Information
Due to you potentially having taken a break from physical activity at some point during your pregnancy and early postpartum, it is important to address any de-conditioning all over the body. If you have had a caesarean delivery, ensure you work slowly through the abdominal exercises, listen to your body and allow healing time. Do not swim until scar healing and lochia has finished, usually ~8 weeks. Scan the QR codes for guidance on these exercises.
1. How could this be presented?
2. Could you follow something like this?
3. What guidance is needed?
4. What's missing?
Addition of individual area pages of programme

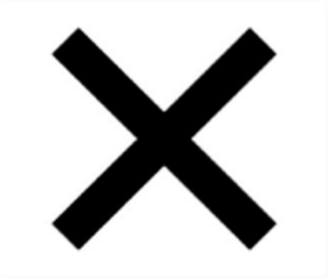

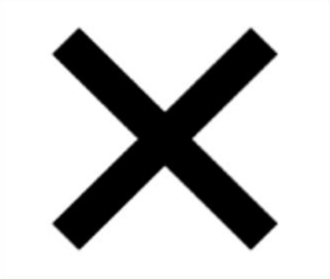

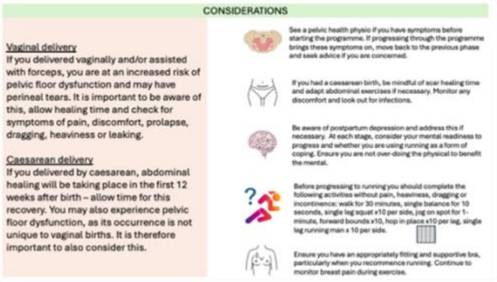

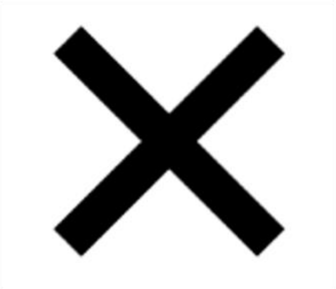

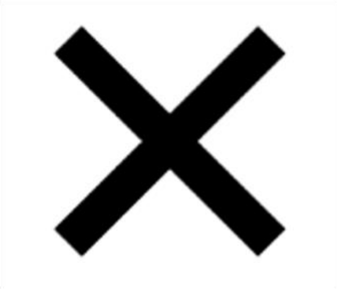

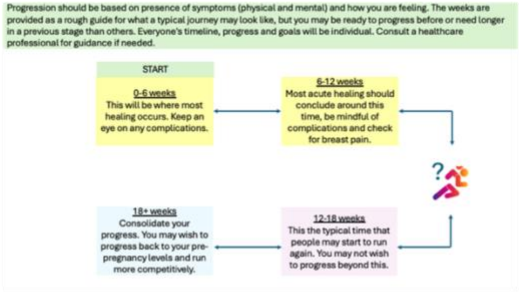

Addition of considerations diagram and progression flow chart

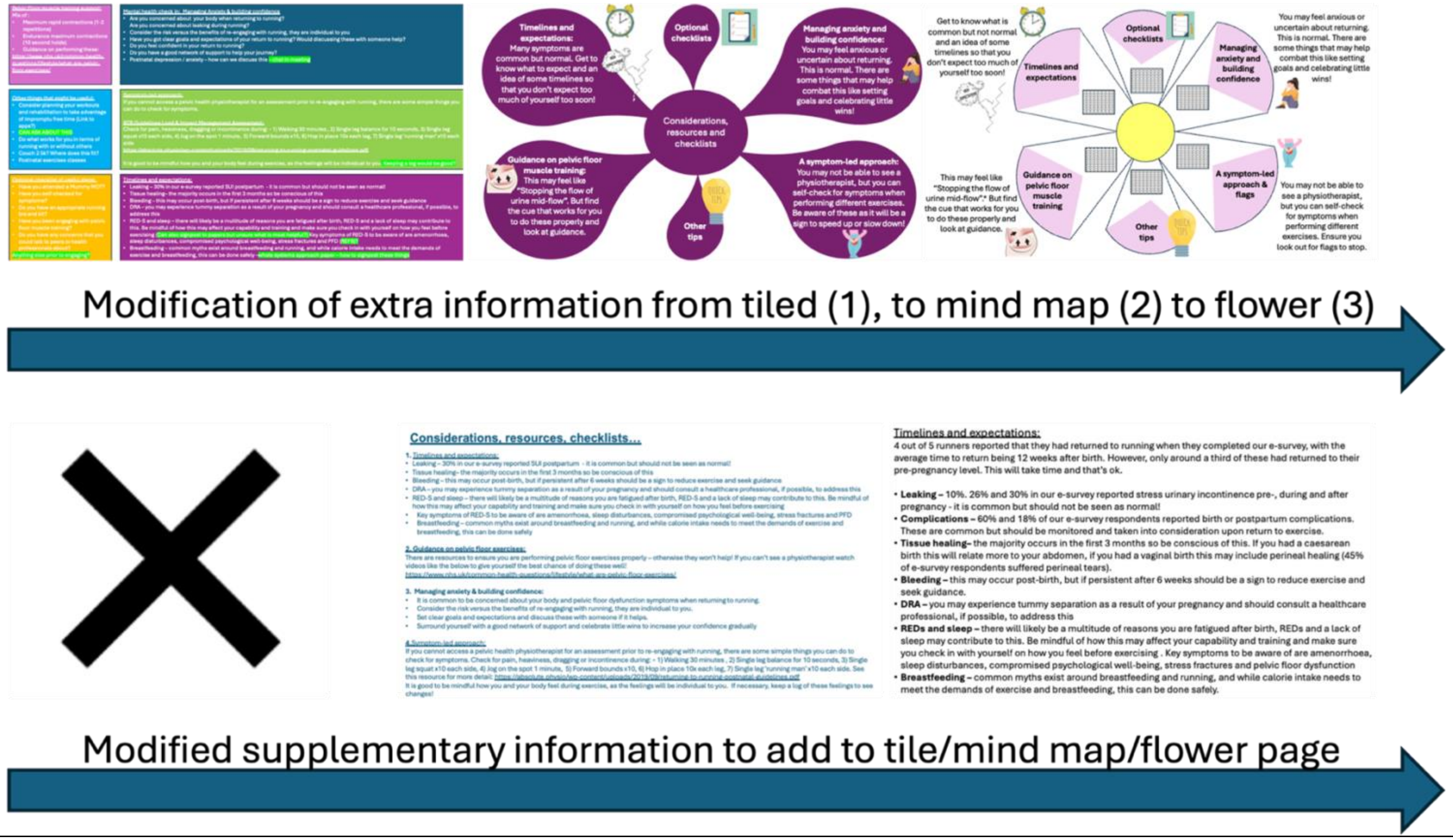

Supplementary Figure 2. Development of the first iteration of the intervention to be presented to the Advisory Group in Chapter 7 from version 1 (left) to 3 (right). Note: The arrow indicates the changes made across the process. 'X' is used to indicate that slide did not exist in the previous or following version. CMO guidelines = Chief Medical Officer guidelines.
